# Chitosan Derivatives with Mucoadhesive and Antimicrobial Properties for Simultaneous Nanoencapsulation and Extended Ocular Release Formulations of Dexamethasone and Chloramphenicol Drugs

**DOI:** 10.3390/pharmaceutics12060594

**Published:** 2020-06-26

**Authors:** Aikaterini Karava, Maria Lazaridou, Stavroula Nanaki, Georgia Michailidou, Evi Christodoulou, Margaritis Kostoglou, Hermis Iatrou, Dimitrios N. Bikiaris

**Affiliations:** 1 Department of Chemistry, National and Kapodistrian University of Athens, Panepistimiopolis, Zografou, 15771 Athens, Greece; katerina_karava1995@hotmail.com; 2 Laboratory of Chemistry and Technology of Polymers and Dyes, Department of Chemistry, Aristotle University of Thessaloniki, GR54124 Thessaloniki, Greece; marlazach@chem.auth.gr (M.L.); sgnanaki@chem.auth.gr (S.N.); michailidougeorgia18@gmail.com (G.M.); evicius@gmail.com (E.C.); 3 Laboratory of Chemical and Environmental Technology, Department of Chemistry, Aristotle University of Thessaloniki, GR-54124 Thessaloniki, Greece; kostoglu@chem.auth.gr

**Keywords:** chitosan, derivatives, mucoadhesion, antimicrobial activity, ocular release, nanoencapsulation, dexamethasone sodium phosphate, chloramphenicol, sustained release

## Abstract

The aim of this work was to evaluate the effectiveness of neat chitosan (CS) and its derivatives with 2-acrylamido-2-methyl-1-propanesulfonic acid (AAMPS) and [2-(methacryloyloxy)ethyl]dimethyl-(3-sulfopropyl)ammonium hydroxide (MEDSP) as appropriate nanocarriers for the simultaneous ocular administration of dexamethasone sodium phosphate (DxP) and chloramphenicol (CHL). The derivatives CS-AAMPS and CS-MEDSP have been synthesized by free-radical polymerization and their structure has been proved by Fourier-Transformed Infrared Spectroscopy (FT-IR) spectroscopy. Both derivatives exhibited low cytotoxicity, enhanced mucoadhesive properties and antimicrobial activity against *Staphylococcus aureus* (*S.aureus*) and *Escherichia coli* (*E. coli*). Encapsulation was performed via ionic crosslinking gelation using sodium tripolyphosphate (TPP) as the crosslinking agent. Dynamic light scattering measurements (DLS) showed that the prepared nanoparticles had bimodal distribution and sizes ranging from 50–200 nm and 300–800 nm. Drugs were encapsulated in their crystalline (CHL) or amorphous (DexSP) form inside nanoparticles and their release rate was dependent on the used polymer. The CHL dissolution rate was substantially enhanced compared to the neat drug and the release time was extended up to 7 days. The release rate of DexSP was much faster than that of CHL and was prolonged up to 3 days. Drug release modeling unveiled that diffusion is the main release mechanism for both drugs. Both prepared derivatives and their drug-loaded nanoparticles could be used for extended and simultaneous ocular release formulations of DexSP and CHL drugs.

## 1. Introduction

Most of the common eye diseases are treated with eye drops where drugs have been dissolved, since they are the simplest, safe and easy-to-use formulations [1]. These drops are aqueous solutions or oily suspensions, but, due to eye reflection, blinking and tear fluid turnover, it is estimated that only about 5% of the administered drug penetrates the cornea [2,3,4,5]. Moreover, only a limited volume of up to 30 μL of the eye drop solution can be applied onto the ocular surface without overflow, reducing the drug administration even further [6]. All of the above factors lead to poor drug effectiveness, and, in order to overcome this, eye formulations must contain highly concentrated drug amounts, which can, however, cause severe side-effects, with the damage of eye cellular surface to be the most serious [7,8,9].

Mucoadhesive drug delivery systems (DDS) are very important since they are able to deliver a drug directly to a specific organ, and thus allow targeting, increase the on-site drug concentration and prolong the drug administration enhancing its bioavailability and effectiveness [10,11,12]. Several mucoadhesive polymers have already been recognized as promising matrices for drug delivery, not following the usual routs of administration, but the transmucosal routes. For that, they must be able to adhere to the mucosa. This ability arises from their property to interact with the mucosal membrane by several ways, including physical bonding, such as interfusion or interpenetration into the mucus layer, or secondary chemical interactions, mainly ionic interactions or hydrogen bond formation. Regarding the latter, the selected polymers must contain appropriate reactive groups, such as carboxyl (–COOH), amino (–NH_2_), hydroxyl (–OH), sulfate (–SO_3_H), etc.

Chitosan is such a polymer, since, due to its functional amino and hydroxyl groups, it presents inherent mucoadhesive properties compared to other natural polymers, such as starch or cellulose, meaning that, in combination with its increased membrane permeability, it is an ideal polymer to produce mucoadhesive DDS. Chitosan (CS) micro- and nanoparticles or micelles have been proved effective as drug carriers, since they are easily prepared, they offer high loading efficiency and reduced side effects, and mainly due to their valued aforementioned mucoadhesivity that leads to an extended drug release [13,14,15]. However, CS also has several drawbacks, such as limited mucoadhesive strength, low chemical purity and swelling properties, as well as low water solubility in neutral pH. To adjust these, various attempts to produce chitosan derivatives with appropriate monomers containing several reactive groups have been made [16,17,18]. Attractive cases of such derivatives are trimethyl chitosan (TMC), which has a cationic charge (–N^+^(CH_3_)_3_) groups attached on its backbone [19,20], carboxymethyl chitosan, which, due to the existence of both carboxyl and amino groups, forms strong mucoadhesion interactions with mucin [21,22] and thiolated CS derivatives, which can form covalent bonds with cysteine-rich subdomains of mucus glycoproteins [23,24,25]. In the present work, CS derivatives containing tertiary amino groups and sulfonic groups have been chosen to be tested as appropriate matrices for the simultaneous encapsulation and extended ocular release of chloramphenicol and dexamethasone drugs.

Chloramphenicol is a broad-spectrum antibiotic against most Gram-negative and Gram-positive, as well as anaerobic, bacteria. It is used to treat infectious conjunctivitis in the form of eye drop solutions. Its primary effect is to inhibit the synthesis of prokaryotic protein and it can be used in wound dressings [26], for the preparation of antimicrobial films with nano-engineered three-dimensional hierarchical surfaces by nanoimprint lithography [27], as a localised drug delivery system encapsulated in β-pyrophosphate for tissue engineering applications [28] and eye infections treatment. Due to its extremely low water solubility (1:400), it is not possible to prepare eye drop solutions with chloramphenicol concentrations higher than 0.5–1%, a fact that drastically reduces its bioavailability and thus its effectiveness [29]. This already low concentration is even further reduced in the conjunctival sac due to the lacrimal drainage system. To achieve an appropriate treatment chloramphenicol of at least 2.5% should be administered daily to the eye for about 5–6 days [30]. Poor drug solubility is maybe the greatest challenge in the field of pharmaceutical technology and drug dispersion in a polymer matrix is by far the most effective solution.

For the production of eye drop solutions, chloramphenicol is usually slightly heated in buffer solutions containing boric acid/borax in order to form sodium salt complexes, but that does not particularly increase its solubility [29]. Several other approaches can be found in literature, such as the treatment with cyclodextrins (CDs), where the aromatic part of chloramphenicol enters the cyclodextrin cavity, forming amorphous complexes with subsequently increased solubility [31,32]. Additional compounds, such as boric acid/borax (1:54 water-soluble) [29] or amino acids [33] can also be added to this system to further enhance its solubility. In all cases, drug release was completed after 5–60 min, depending on the type of the prepared complex. Chitosan derivative with acrylic acid (CS-AA) as well as their copolymers with *N*-isopropyl acrylamide (NIPAM) or 2-hydroxyethyl methacrylate (HEMA) have been used for chloramphenicol encapsulation in the form of nanoparticles [34]. 

Dexamethasone (Dex) is one of the most widely used corticosteroid drugs for a vast variety of ocular conditions, including anterior chamber inflammation, and for the treatment of various other inflammatory disorders with high potency and effectiveness, since its anti-inflammatory activity is thirtyfold greater than that of cortisol [35,36]. Its function has been ascribed to inhibit the proteins of phospholipase A2 group, and several innovative dexamethasone pharmaceutical systems, including hydrogels, nano-sponges, nano-micelles, dendrimers, implants, micro and nanoparticles, have been prepared to treat ocular diseases [37]. However, dexamethasone, due to its bulkier and hydrophobic structure, has poor water solubility, and, for this reason, its derivative, dexamethasone sodium phosphate (DexSP), has been extensively used instead for ocular treatment diseases (e.g., uveitis, injury) via topical administration [38,39]. It displays higher water solubility, but, in the form of eye drop solutions, it is quickly eliminated within 5 min after topical administration [40,41]. Commercial DexSP eyedrops are, therefore, generally inadequate and demand frequent administration to maintain an effective therapeutic concentration. Hence, advanced delivery systems are again required in order to extend its release.

In such an attempt, carboxymethyl chitosan derivative was used to prepare nanocomposites with layered double hydroxide (LDH) as a sustained-release and prolonged precorneal retention formulation of DexSP [42]. From in vitro ocular tissue distribution on rabbits, it was found that the bioavailability of prepared nanocomposite eye drops was significantly improved compared to the commercial products. Chitosan oligosaccharide-valylvaline-stearic acid nano-micelles were also designed for the topical ocular drug delivery of Dex and it was found that all nano-micelles exhibited sustained release rates [43]. In a recent study, a series of dexamethasone-glycol chitosan (Dex-GCS) conjugates have been prepared for dexamethasone administration in the form of nanoparticles [44]. It was found that CS-glycol nanoparticles have mucoadhesive properties and Dex release was progressive up to 8 h, followed by a plateau up to 48 h, at which point only 80% of the total drug was released and the other amount remained entrapped in the nanoparticles. Furthermore, these nanoparticles showed longer precorneal retention compared to the aqueous solutions.

Taking all that has previously been discussed into account, the aim of the present work is to enhance the solubility of CHL and to prepare sustained release drug formulations with enhanced mucoadhesion properties for both CHL and DexSP drugs. For this reason, CS derivatives with appropriate groups, such as tertiary amino groups and sulfonic groups, that could improve the mucoadhesive and antimicrobial properties of CS have been synthesised. These materials will be used for the nanoencapsulation of chloramphenicol and dexamethasone sodium phosphate drugs to obtain sustained and extended ocular release formulations for simultaneous administration. Such formulations have gained great attention in recent years due to their ability to simultaneously treat microbial infections and inflammatory eye diseases. Chitosan and its derivatives in the form of nanoparticles have been chosen, since they offer additional advantages as ocular drug delivery platforms. These include: improved drug stability and solubility, adjustable surface properties and increased adhesion, controlled drug release, as well as enhanced drug penetration and permeation through the mucus membrane [45].

## 2. Materials and Methods

### 2.1. Materials

Chitosan (CS) of medium molecular weight (190–310 kDa, degree of deacetylation 75–85%), 2-acrylamido-2-methyl-1-propanesulfonic acid (AAMPS) and sodium tripolyphosphate (TPP) used as ionic crosslinker, were purchased from Sigma-Aldrich Co. (Steinheim, Germany). 2-(methacryloyloxy)ethyl]dimethyl-(3-sulfopropyl)ammonium hydroxide (MEDSP) (purum 95%) was purchased from Fluorochem (London, United Kingdom), while potassium persulfate (K_2_S_2_O_8_, KPS, purum ≥ 99.0%) was purchased from Merk (Athens, Greece). Dexamethasone sodium phosphate (DexSP) and chloramphenicol (CHL) drugs with purity >99.99% were purchased from Pharmathen S.A. (Athens, Greece). All other reagents and solvents used were of analytical grade.

### 2.2. Synthesis of CS Derivatives

**Scheme 1:**
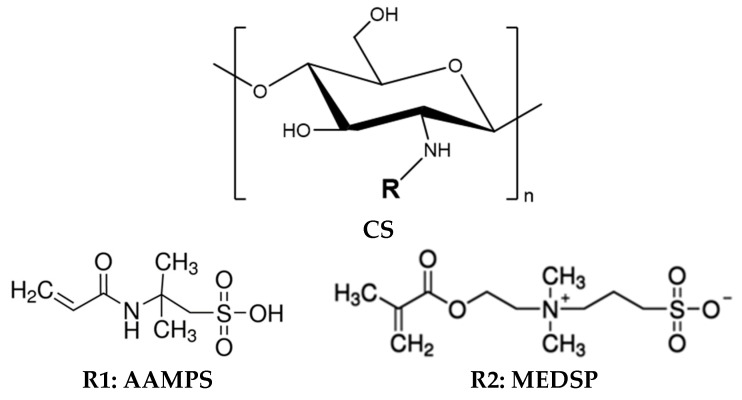
Chemical structures of prepared CS derivatives (CS-AAMPS and CS-MEDSP) prepared with AAMPS and MEDSP monomers, respectively.

Grafted CS with MEDSP and AAMPS monomers (CS-MEDSP and CS-AAMPS, respectively) were synthesized via free-radical polymerization as was previously reported [46]. In brief, 10 g of CS (62 mmol monosaccharide residues) was dissolved in 400 mL of aqueous acetic acid solution of 2% v/v concentration. The mixture was left under magnetic stirring at room temperature. After complete chitosan dissolution, 1.5 g of MEDSP (5.4 mmol) or AAMPS (7.2 mmol) and 37.5 mg of KPS were added to the mixture. The grafting reaction took place at 60 °C, for 2 h, under nitrogen atmosphere and continuous magnetic stirring, leading to the formation of a viscous liquid. The product was then precipitated by an aqueous solution of NaOH (1 M in concentration). The resulting precipitate was collected, frozen and freeze-dried at −108 °C. The final products were further purified by Soxhlet using methanol as an eluent. Their structures appear in Scheme 1.

The grafting percentage (GP) was calculated based on the following equation:

GP = (M_fin_ − M_in_)/M_in_ × 100% (1)

where M_in_ and M_fin_ are the mass of chitosan before and after the grafting process, respectively.

### 2.3. Preparation of CS Nanoparticles

**Scheme 2:**
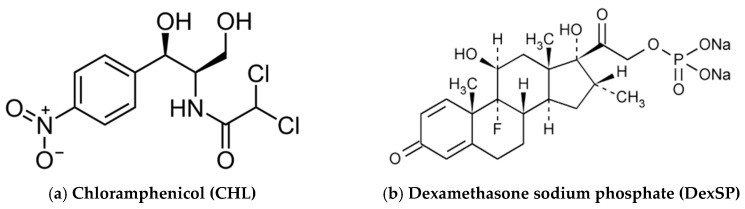
Chemical structures of the used drugs for ocular release formulations. (**a**) Chloramphenicol (CHL), (**b**) Dexamethasone sodium phosphate (DexSP).

CS nanoparticles loaded with chloramphenicol and dexamethasone sodium phosphate drugs (Scheme 2) were prepared according to the ionotropic gelation method using TPP as an ionic crosslinking agent [47]. Neat CS nanoparticles were obtained upon the addition of TPP aqueous solution to a CS acetic acid solution (pH about 4.5) in a CS/TPP ratio of 4/1 *w*/*w* (1.6 g CS and 0.4 g TPP in 100 mL solution) and a stirring rate of 800 rpm. For the preparation of drug-loaded nanoparticles, the same conditions, as described before, have been used. DexSP or CHL (0.5 g of DexSP and 1 g of CHL) were previously diluted in ethanol and were then added dropwise in the CS or its derivatives’ aqueous solutions under and a stirring rate of 800 rpm before adding the crosslinking agent to prepare the nanoparticles. Every sample was prepared in triplicate and the results represent the average value. The non-entrapped drug and the remaining dissolved CS or its derivatives were removed by ultra-centrifugation at 13,000 rpm for 20 min. The precipitated nanoparticles were washed twice with fresh ultrapure water, again centrifugated and collected in the form of an aqueous suspension. They were then frozen and lyophilized by a freeze-drier system (Scanvac, Coolsafe 110-4 Pro, Labogen Scandinavia) for 24 h at −108 °C to obtain dried nanoparticles. 

The % yield of nanoparticles was calculated based on the following equation:

Yield (%) = [Weight of nanoparticles/Initial weight of polymer and drug] × 100(2)

### 2.4. Characterization of Chitosan Derivatives

#### 2.4.1. Fourier-Transformed Infrared Spectroscopy (FTIR)

FT-IR spectra were obtained on a Perkin-Elmer FT-IR spectrometer (Spectrum 1, Waltman, MA, USA) using pellets of CS and its derivatives in KBr. Spectra were obtained in the range of 4000 to 400 cm^−1^, at a resolution of 4 cm^−1^, using 16 co-added scans. All spectra presented are baseline corrected and normalized.

#### 2.4.2. Wide angle X-Ray Scattering

X-ray powder diffraction (XRD) patterns were recorded using an XRD-diffractometer (Rigaku-Miniflex II, Chalgrove, Oxford, UK) with a CuK_α_ radiation for crystalline phase identification (λ = 0.15405 nm for CuK_α_). The sample was scanned from 5 to 50°.

#### 2.4.3. Swelling Study

Swelling ability was evaluated by measuring the water sorption capacity in water (pH = 7.0) for CS, CS-AAMPS and CS-MEDSP as was previously described [26]. Initially, each sample was carefully weighed (W_0_) and immersed in each solution separately for several hours. At predetermined time intervals, the grafted derivatives were removed from the solutions, wiped off by filter paper in order to remove the excess surface water, and weighed again in order to determine the swelling weight (W_n_). The percent weight change of the samples during the swelling experiment (i.e., cumulative weight changes due to matrix swelling, S(ti)%) was calculated by the following equation:

S(ti)% = [(W_n_ − W_0_)/W_0_] × 100 (3)

All measurements were performed in triplicate.

#### 2.4.4. 3-[4,5-Dimethylthiazole-2-yl]-2,5-Diphenyltetrazolium Bromide (MTT) Assay

In order to assess the cytotoxicity levels of the tested materials, Adipose tissue-derived mesenchymal stem cells (ASC) were exposed to them and the MTT assay was performed (Sigma-Aldrich) 24 after the initial cell plating. Day 0 was defined as the day of the materials’ addition in the culture supernatants in two different concentrations: 100 and 500 μg/mL in Dulbecco’s Modified Eagle Medium (DMEM). One day before the addition, the cells were seeded on plastic surfaces of 24-well plates at a density of 30,000 cells in 500 μL cell culture medium (Dulbecco’s modified Eagle’s medium supplemented with 10% Fetal Bovine Serum and 2% Penicillin/Streptomycin) per well for their initial adherence. The tested materials were sterilized under decreasing concentrations of ethanol (100%, −70%, −50%), washed with distilled water and dried overnight under sterile conditions. Untreated cells were used as a control group in the same cell number as the other groups. Briefly, upon 24 h of ASCs culture in the presence of materials, and after the medium removal from the wells, MTT reactant was introduced in a ratio of 1:10 in DMEM culture medium and was followed by a 4-h incubation in 37 °C with 5% CO_2_. Upon the removal of the MTT, 1 mL/well of DMSO was introduced for an additional hour of incubation in the same conditions. The reduction of MTT was counted in 570 and 630 nm wave length (Perkin Elmer).

#### 2.4.5. Mucoadhesive Strength

Adhesive force measurements were performed at room temperature (25 ± 1 °C) using an Instron 1122 dynamometer (Norwood, MA, USA) containing a 10 kg full-scale load cell, as was described in our previous work, but with a small variation [48]. For this reason, films with sizes 2 × 2 cm (4 cm^2^) have been prepared from each polymer by solution casting (4 *wt*% of each polymer was dissolved in acetic acid solution, pH < 4.5). A piece of fresh pig intestinal mucosa, with similar sizes as the prepared polymers films (2 × 2 cm), was fixed with an epoxy adhesive on a Teflon support that was placed on the stable cell grid of instrument. In the same way polymeric films have also been fixed on a Teflon support and immersed into a phosphate buffer solution (pH = 7) for 5 min for wetting, and the support was afterwards placed in the moving cell grid of the instrument. After both cell grips were placed in the instrument, the grid with the fixed polymeric film was moved slowly to the fresh pig intestinal mucosa surface, and a pressure of 0.2 kg/cm^2^ was applied for 5 min. The mucoadhesive strength was calculated from the force that was recorded while detaching the film from the mucosa surface, using a crosshead speed of 15 mm/min. Each value corresponds to the average of three different measurements of the same sample.

#### 2.4.6. In Vitro Antibacterial Activity Testing

The antibacterial activity of synthesized derivatives (1.75 *wt*% in acetic acid solution) was evaluated via the agar diffusion method given in a previous article of ours [49]. In brief, the inhibition zones were measured on Mueller Hinton agar plates inoculated with *Staphylococcus aureus* (*S.aureus*) and *Escherichia coli* (*E. coli*). Colonies isolated from nutrient agar were inoculated to the nutrient broth and further incubated at 37 °C for 18–24 h. The bacterial cultures were transferred to Muller Hinton Broth (Steinheim, Germany) for a 2-h incubation and the inoculums were adjusted at McFarland 0.5 standard. After each 0.1 mL inoculum spreading, some amount of the diluted materials (0.05 mg/mL) was placed on Mueller Hinton agar. The plates were incubated at 37 °C for 24 h and the diameters of the inhibition zones around the CS and its derivatives were measured and compared with that of blank samples. Each test was repeated three times.

### 2.5. Characterisation of Encapsulated Nanoparticles in CS Derivatives

#### 2.5.1. Scanning Electron Microscopy

Scanning electron microscopy (SEM) micrographs of prepared nanoparticles were performed with an electron microscope (JMS-840, Peabody, MA, USA). The freeze-dried samples were covered with carbon coating in order to avoid charging under the electron beam. The operating conditions were the following: accelerating voltage 20 kV, probe current 45 nA, and counting time 60 s.

#### 2.5.2. Size Measurements of Nanoparticles

The particle size distribution of prepared CS-derivatives/drug encapsulated nanoparticles was determined by dynamic light scattering (DLS) using a Zetasizer Nano Instrument (Malvern Instruments, NanoZS, ZEN3600, UK) operating with a 532 nm laser. A suitable amount of nanoparticles was dispersed in distilled water, creating a total concentration of 1%, and was kept at 37 °C under agitation at 100 rpm.

#### 2.5.3. Differential Scanning Calorimeter (DSC)

The differential scanning calorimeter (DSC) study was performed using a Perkin-Elmer, Pyris Diamond DSC (Waltham, MA, USA), calibrated with Indium and Zinc standards. Dried samples (24 h at 100 °C under vacuum) of 10.0 ± 0.1 mg sealed in aluminum pans were heated from room temperature until 275 °C at a heating rate of 20 °C/min under nitrogen atmosphere. 

#### 2.5.4. Drug Loading 

For the determination of the drug loading, 10 mg of the prepared drug loaded (CHL or DexSP) nanoparticles were dissolved in 10 mL of aqueous acetic acid solution (2% *v*/*v* in concentration):methanol 80:20 in ratio and the resultant solution was analysed for assay. Drug loading (DL) was calculated from the following equations:

DL (%) = [weight of drug in nanoparticles]/[weight of nanoparticles] × 100(4)

EE (%) = [weight of drug in nanoparticles]/[initial weight of drug] × 100(5)

#### 2.5.5. In Vitro Dissolution Studies

For the in vitro release studies, DISTEK Dissolution Apparatus Evolution 4300 (2100C) (North Brunswick, NJ, USA), equipped with an autosampler using the basket method (USP I method), was used. A sponge was placed to each dissolution vessel corresponding to approximately 400 mg of each formulation in an appropriate transdermal patch holder, with its application side up. The test was performed at 37 ± 1 °C with a rotation speed of 50 rpm. The dissolution medium was 1000 mL of phosphate-buffered saline (PBS)-simulated body fluid, pH = 7.4. Two (2) mL of the aqueous solution was withdrawn from the release media and analyzed for drug content (CHL or DexSP) with the aid of a Shimadzu HPLC system (Kyoto, Japan). The column used was a Canada Newswire (CNW) Technologies Athena C18, 120 A, 5 μm, 250 mm × 4.6 mm at column temperature of 25 °C. The mobile phase consisted of ACN/H_2_O (acidified with phosphoric acid at final pH = 5) 40/60 *v*/*v*, and the flow rate was adjusted to 1.0 mL/min. Concentration determination was performed using an HPLC-UV (Kyoto, Japan) apparatus at 278 nm and was based on a previously created calibration curve and at 254 nm for DexSP. The injection volume was 10 μL. The calibration curve was created by diluting a stock methanol solution of 100 ppm CHL to concentrations 0.5, 1.0, 2.5, 5.0, 10.0, 20 and 25 ppm using the mobile phase. The calibration curve for DexSP was created by diluting a stock methanol solution of 100 ppm to concentrations 0.01, 0.05, 0.1, 0.25, 0.5, 1.0, 2.5, 5.0, 10.0 and 20 ppm using water.

## 3. Results and Discussion

### 3.1. Synthesis of CS Derivatives

CS derivatives were synthesized by free-radical polymerization. The molar ratio was 1/11.5 mol/mol MEDSP/CS and 1/8.6 mol/mol AAMPS/CS, meaning that 1 mol of MEDSP or AAMPS corresponds to 11.5 or 8.6 mol of monosaccharide residue of CS, respectively. This low degree of derivatization was chosen in order not to consume all amino groups of CS during polymerization, but instead retain enough free –NH_2_ onto CS. These groups are necessary for CS to maintain its mucoadhesive properties. Moreover, due to the low molar ratio of the used AAMPS and MEDSP monomers, the side chains on CS’s backbone that consisted of AAMPS and MEDSP macromolecules did not form. The synthesis of /CS-MEDSP derivative was reported also in literature [50,51] but for a different kind of application than in our study, while CS-AAMPS derivative is synthesised for the first time. The chemical structures of the prepared derivatives are shown in Scheme 1. The reaction yield, as was calculated by weight measurements after Soxhlet extraction, was estimated at 63% for MEDPS and 82% for AAMPS. According to these, it is clearly indicated that CS-MEDPS and CS-AAMPS derivatives of almost similar molar ratios of monosaccharide units to grafted monomers 7.245/1 and 7.052/1 mol/mol, respectively, have been prepared. It seems that the existence of the methyl group in MEDPS slightly reduces its reactivity, compared to the AAMPS monomer. This was proved from our preliminary work, and for this reason a higher molar ratio between MEDPS/monosaccharide units was initially used (1/11.5 mol/mol) than in the case of AAMPS/monosaccharide units (1/8.6 mol/mol), in order to prepare derivatives with an almost similar degree of substitution. This strategy successfully led to the preparation of CS derivatives of similar CS units/grafted monomers’ molar ratios. Thus, any differences to their properties should be consequently attributed to their different chemical structure and not their slightly different degree of substitution.

**Figure 1:**
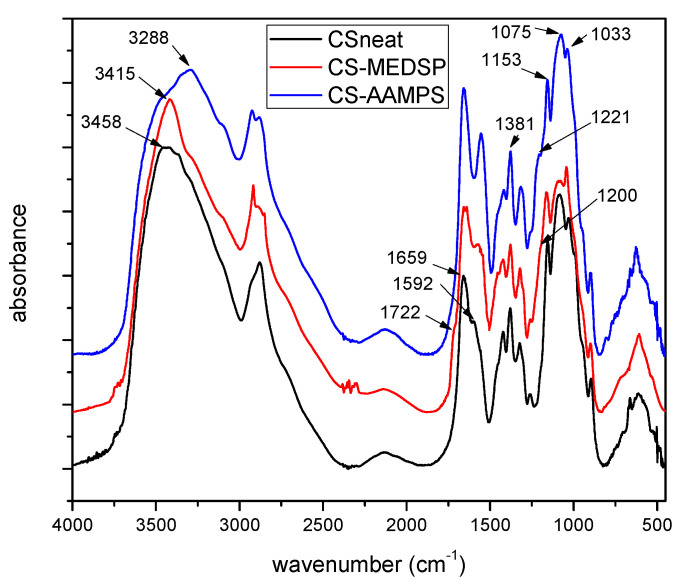
FT-IR spectra of neat CS and its derivatives with AAMPS and MEDSP.

In order to examine the successful synthesis of grafting to CS, the FT-IR spectra of neat CS, MEDSP and AAMPS were obtained. As can be seen in Figure 1, CS showed all its characteristic peaks, i.e., a broad peak at 3458 cm^−1^, owing to the hydroxyl groups of CS, two shoulders recorded at 3259 and 3088 cm^−1^ due to its primary and secondary amino groups, the peaks at 1659 and 1592 cm^−1^ are attributed to absorption amide I and II, respectively, and a peak at 1381 cm^−1^ characteristic of the –CH_2_ bending. There are also additional bands at 1153cm^−1^ owing to the asymmetric stretching of the C–O–C bridge, and two bands at 1075 and 1033 cm^−1^ attributed to the skeletal stretching vibration of CO, all characteristic of chitosan’s saccharide structure [52]. AAMPS monomer recorded a sharp peak at 3288 cm^−1^ characteristic of the >NH symmetric stretching, a peak at 1665 cm^-1^ owing to the CO–NH amide stretching, a peak at 1613 cm^−1^ attributed to the =CH vinyl group and three peaks at 1245, 1129 and 1085 cm^−1^ corresponding to the O=S=O symmetric stretching, C–O–C stretching, and C–N stretching, respectively. The MEDSP monomer also recorded its expected characteristic peaks; a peak at 1722 cm^−1^ assigned to the stretching vibrations of C=O ester bond, a “shoulder” peak at 1636 cm^−1^ attributed to the stretching vibration of =CH vinyl group, a peak at 1293 cm^−1^ due to C–N, and the symmetrical and asymmetrical stretching vibrations of S=O bond at 1039 and 1187 cm^−1^, respectively.

In the spectrum of CS-AAMPS derivative, a peak of the –OH groups at 3542 cm^−1^ is observed, two strong peaks at 3272 and mainly at 3084 cm^−1^ characterizing the primary and secondary amino groups vibrations, respectively, a strong peak 1657 cm^−1^ due to the CO–NH amide vibration, and also a strong peak at 1221 cm^−1^ due to the existence of sulfonic groups. Comparing the positions and intensities of the main peaks of neat CS and CS-AAMPS, some clear shifts can be noticed. Moreover, the strong peak intensity at 3084 cm^−1^ (secondary amino groups) and the appearance of new peaks at 1657 cm^−1^ and 1221 cm^−1^ in the case of CS-AAPMS derivative, are clear indications of the incorporation of AAMPS monomer onto the CS backbone. Likewise, in the spectrum of CS-MEDSP derivative, there is a recorded “shoulder” peak at 3433 cm^−1^, due to the vibrations of –OH groups of CS, a strong absorption at 3355 and 3296 cm^−1^, due to the hydrogen bonded –OH groups and primary amino groups, respectively, a low intensity peak recorded as a shoulder at 3086 cm^−1^ owing to secondary amino groups, a small peak at 1720 cm^−1^ (ester group) and a stronger at 1654 cm^−1^ (due to CS absorption), a shoulder at 1200 cm^−1^ (SO_3_^−^ group) and a high intensity peak at 1031 cm^−1^ (C–N absorption). All these and particularly the absorptions at 1720 and 1200 cm^−1^ prove the successful synthesis of CS-MEDSP derivative. Unfortunately, tertiary amines are not associated with any unique absorption peaks and thus are difficult to be detected using FT-IR spectroscopy [53]. According to the above discussed spectra, it is clear that the desired CS derivatives have been successfully prepared, and that some interpolymeric interactions, such as hydrogen bonding between the additional reactive groups of AAMPS (–CO–NH and –SO_3_H) and MEDSP (–CO–O and SO_3_^−^) and the –NH_2_ and –OH groups of CS, have also occurred. Similar interactions have been reported in CS derivatives with NIPAM and HEMA [54].

**Figure 2:**
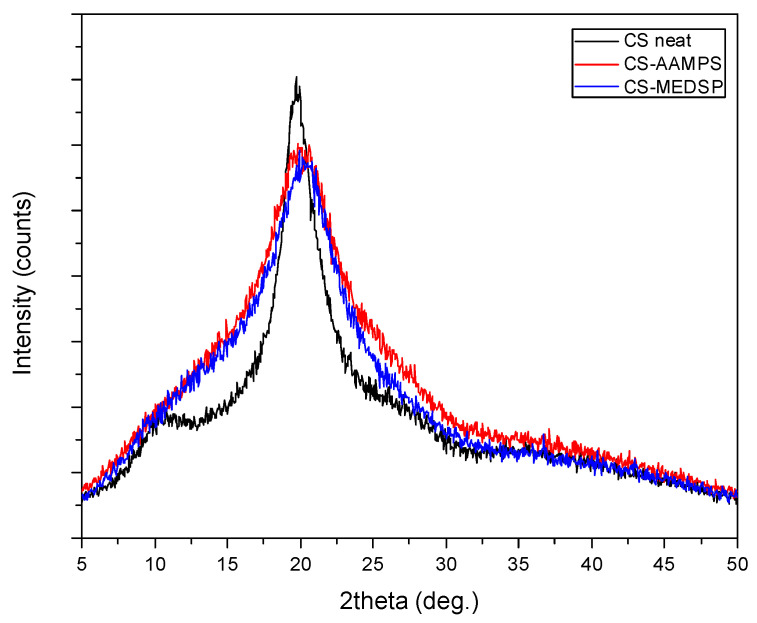
XRD patterns of neat CS and its derivatives with AAMPS and MEDSP.

CS is a semi-crystalline polymer, as all polysaccharides, due to the strong hydrogen bonds between its hydroxyl groups, as well as the ones between the amino and hydroxyl groups. This was proved by the obtained XRD patterns where neat CS recorded two broad halo peaks at 2θ = 10.5 and 19.8°. As can be seen in Figure 2, the prepared CS-AAMPS and CS-MEDSP grafted derivatives are also semi-crystalline since a strong peak at 20.3° was recorded in their patterns. Both AAMPS and MEDSP monomers are crystalline materials. However, it becomes evident that the degree of crystallinity was reduced, since the intensity of the recorded peak at 20.3° is lower than that of neat CS peak and the curve is also broadened. Furthermore, the low intensity peak at 10.5° was not recorded in derivatives. The introduction of side groups in CS’s macromolecular chains is well known that reduces their ability to fold and create crystallites, thus producing less crystalline [55,56] or completely amorphous derivatives [50]. This also occurs in the case of the produced CS-AAMPS and CS-MEDSP derivatives.

**Figure 3:**
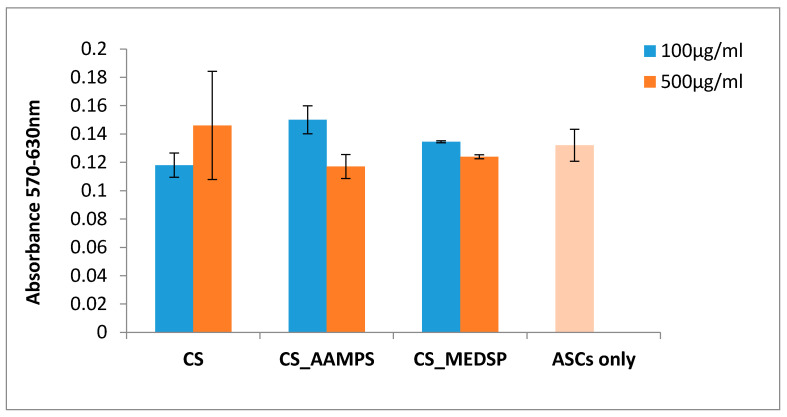
Cells viability was determined by MTT assay cells’ treatment with our samples for 24 h. The absorbance values are directly proportional to metabolic activity of the cells.

CS is a biocompatible polymer and can be used in biomedical and pharmaceutical applications. However, since there are no existing toxicity tests for these particular prepared derivatives, an MTT test was performed to ensure that they are safe to use for drug nanoencapsulation and ocular applications. In Figure 3, the Y’Y axis’ values represent the reduction of yellow 3-(4,5-dimethythiazol2-yl)-2,5-diphenyl tetrazolium bromide (MTT) by mitochondrial succinate dehydrogenase. The MTT enters the cells and passes into the mitochondria, where it is reduced to formazan product. The cells are then solubilized with an organic solvent and the released, solubilized formazan reagent is measured spectrophotometrically. Since the reduction of MTT can only occur in metabolically active cells, the level of activity is a measure of the viability of the cells. A concentration of 100 or 500 mg/L of each polymer was used, and, as can be seen in Figure 3, both derivatives have similar absorbances with neat CS, indicating that these are biocompatible materials with low toxicity [57,58]. In all cases, the recorded values are very close to that of the reference sample where no material was added on the ASCs. Similar results have been found also from our group for other CS derivatives containing acrylates, such as poly(2-hydroxyethyl methacrylate) (PHEMA) [59] and 2-hydroxyethylacrylate [49], while the biocompatibility of CS-MEDSP derivative was also proved in a previous study reported in literature [50].

Except for the biocompatibility of CS and its derivatives, their antimicrobial properties against *Staphylococcus aureus* (SS) and *Escherichia coli* have also been examined. It is well known that neat CS exhibits antimicrobial activity since it can disrupt or destabilize the barrier properties of the outer membranes of Gram-negative bacteria or due to its ability to permeate the microbial plasma membrane [60,61,62]. This property can be further enhanced with the addition of appropriate monomers onto its backbone. The quaternization of chitosan can lead to materials with such improved antibacterial properties against several negative and Gram-positive bacteria [63,64,65,66]. It has also been reported that the addition of sulfoxyamine groups onto CS, produces a derivative with enhanced mucoadhesive and antibacterial properties [67]. Therefore, in this work, monomers carrying similar groups, such as tertiary amino groups and sulfoxide, have been chosen.

Table 1 summarizes the inhibition growth zones of each sample against the studied *Staphylococcus aureus* (*S. aureus*) and *Escherichia coli* (*E. coli*) strains, which are the most frequently contacted pathogens. These data have been compared with *neomycin,* which is an aminoglycoside antibiotic used in eyedrops and in many topical medications like ointments and creams. As can be seen, different inhibition zones are observed decanted from the used polymer and also from the different strains. In particular, the antimicrobial activity against the *S. aureus*, proved to be more effective than that against *E. coli*, as indicated by the smaller inhibition zones of *E. coli*. CS has a low antimicrobial activity against both strains. However, comparing the inhibition zones of CS-AAMPS and CS-MEDSP, it is indicated that CS-AAMPS shows slightly lower antimicrobial properties than CS-MEDSP. The antimicrobial properties of derivatives follow the activity of the used monomers. Compared with neomycin it can be seen that the antimicrobial properties of the studied derivatives are smaller against *E. coli* but much better against *S. aureus*. MEDSP seems to have slightly better antimicrobial properties due to its positive charge groups [51]. This was also proved in a previous study comparing neat CS, *N*-trimethyl chitosan (TMC) and *N*-diethylmethyl chitosan (DEMC) inhibition against *S. aureus* [68]. From the recorded data, it was found that TMC has the highest inhibition effect, followed by DEMC, and chitosan has the lowest. This is due to the stronger positive charge of the quaternary ammonium groups found on the derivatives, compared with CS. These groups form strong polyelectrolyte complexes with negative peptidoglycans of the bacterial cell wall, leading to the cell wall disruption and thus the bacterial growth. Another quaternary CS derivative prepared with 2-hydroxypropyltrimethyl ammonium chloride (HACC) showed high antimicrobial activity against *S. aureus* and *E. coli* [69]. Furthermore, it was found that, by increasing the degree of quaternization, the antimicrobial activity was increased too. N,N,N-Trimethyl O-(2-hydroxy-3-trimethylammonium propyl) chitosans (TMHTMAPC) with different degrees of O-substitution were synthesized with enhanced bacteriostatic properties against *E. coli* and *S. aureus* [70].

For prolonged ocular release formulations, the mucoadhesion of used polymers is very important. The mucoadhesion process begins with wetting and gel formation between used polymer and surface of mucus membrane, which is called contact stage, followed by the formation of adhesive interactions via secondary bonds, such as ionic, hydrogen bonds, and week Van der Waals forces with membrane molecules [12,71,72]. These interactions prolonged the polymer adhesion and this stage is called consolidation stage. CS has primary amino groups that, at low pH, can be protonated and these can participate in the electrostatic interactions with the negatively charged sialic acid and sulfate groups of mucin macromolecules [73].

However, the ability of CS amino groups to be protonated is limited to neutral pH conditions, while the formation of a gel structure is also restricted due to its hydrophobicity and semi-crystalline nature. The wettability is maybe the most important stage for a successful and prolonged adhesion of a material or particle on mucus surface and in the case of CS it can be achieved by chemical modifications. This was also proved from a previous work in which it was reported that the mucoadhesive strength of a cellulose derivative depends on its ability to water uptake [74]. The higher this ability is, the more stable the formed gel, and interactions are taking place to a greater extent. For this reason, the swelling behaviour of all derivatives was studied at pH 7, in the form of thin films.

**Figure 4:**
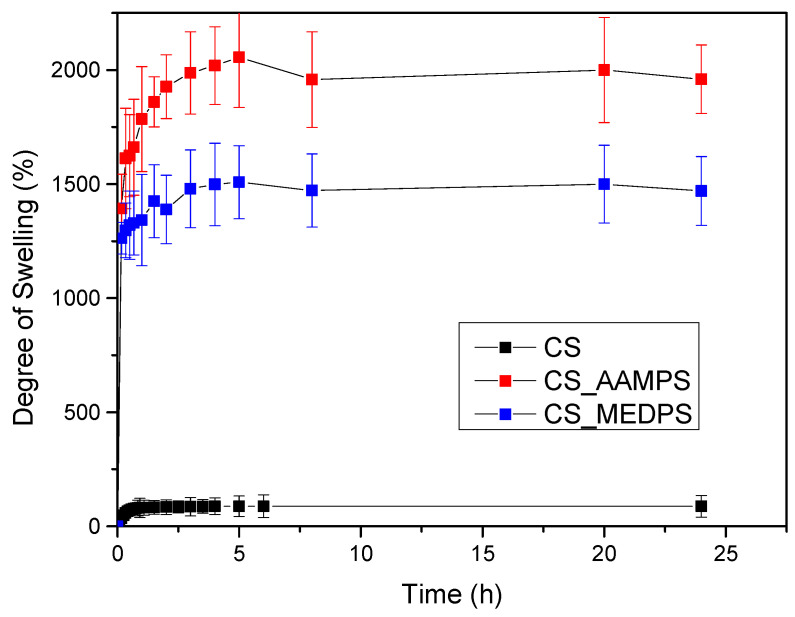
Degree of swelling versus time for CS, CS-AAMPS and CS-MEDPS at pH = 7.

As can be observed in Figure 4**,** chitosan reached its maximum swelling degree in the first hour with a value of 70% at pH = 7 and no further swelling was detected up to 24 h. CS-MEDPS showed a maximum swelling at 5 h, which was estimated at about 1530%, followed by a slow decrease to 1475% in 8 h and then remained almost stable up until 24 h (1469%). Comparable were the results for CS-AAMPS, which reached a maximum swelling value up to 2055% in 5 h, followed by a minor reduction to 1965% after 8 h and then remained stable. It is clearly indicated that both prepared derivatives are more hydrophilic than neat CS, which could be attributed to the additional reactive groups that have been added onto the CS backbone. This is in well agreement with our previous studies in CS derivatives with N-(2-carboxybenzyl)chitosan, which, due to its hydrophilic character because of the added –COOH groups, exhibited a higher degree of swelling than neat CS [75]. Moreover, in acrylic acid/polyethylene glycol composites, it was found that by increasing the acrylic acid content, the swelling degree increases too [76].

To evaluate the mucoadhesive strength of a polymer, several in vitro methods have been applied and most of them are based on mechanical tests used to calculate the detachment work of the formed interface between mucus and polymer [77,78]. From viscoelastic spectra studies, it was found that the gel strength between polymers and mucus can be explained in terms of formation of both physical molecular entanglement and secondary chemical bonds, such as hydrogen bonds [79]. For neat CS, it was found that the detachment force from pig intestinal mucosa depends on the used kind of CS and ranges between 3.9 (low viscosity) and 6.7 (high viscosity) mN/cm^2^ [80]. In the same study, it was found that poly(acrylic acid) has better mucoadhesive properties (11.7 mN/cm^2^) than CS, while other natural polymers, such as pectin, starch and xanthan gum, have no mucoadhesive properties. However, as was already mentioned, the gelling and swelling properties of a polymer are very important. The mucoadhesive strength in the case of our prepared CS derivatives was evaluated using films that have been prepared by solvent casting. In order for a gel to be formed on these films before the attachment with porcine mucosa, they have been activated by being added in water for 5 min. This duration was selected according to Shojaei et al., who evaluated the effect of films’ contact time to mucus surface and found that mucoadhesive strength increases progressively by increasing the contact time from 10 to 300 s [81]. Furthermore, a physical attachment with the mucosa surface is necessary to proceed, and, for this reason, a pressure of 0.2 kg/cm^2^ was applied for 5 min.

From our tensile strength measurements, the mucoadhesive strength of neat CS was estimated at 0.27 N/cm^2^ (Table 2), which is very close to that reported in literature 0.34 N/cm^2^ [82] or 0.42–0.85 N/cm^2^, depending on the type of the used CS [83], while in another study it was found to be 0.58 N/cm^2^ [84]. These values are much higher compared to other polymers like poly(vinyl pyrrolidone), which has a negligible mucoadhesive force (0.006 N/cm^2^) and HPMC (0.157 N/cm^2^) [48].

Both prepared derivatives have much higher mucoadhesive strength, which is dependent on the used side group. CS-AAMPS has a strength of about 0.46 M/cm^2^ and CS-MEDPS of 0.72 N/cm^2^. These values are comparable with polymers with high mucoadhesive strength like poly(acrylic acid) (Carbopol 934) 0.51 N/cm^2^ [85] and other polymers like hydroxyethylcellulose and poly(vinyl alcohol), for which values 0.88 and 5.11 N/cm^2^, respectively, have been reported [84]. Similar to ours, CS methacrylated derivatives were reported to have enhanced mucoadhesive properties [86]. To explain the enhanced mucoadhesive properties of prepared derivatives, the mucoadhesion mechanism of CS should be discussed. Chitosan and mucin in aqueous solutions are known to interact predominantly electrostatically, yielding protein–polysaccharide complexes. Meng-Lund et al. [87] reported that the interactions between chitosan and mucins are stronger at pH = 5.2 than pH = 6.3 and the calculated work force of adhesion was 0.3–0.5 mN and 0.2 mN, respectively, values that are, of course, much lower than those previously mentioned. This is due to the fact that the mucoadhesion of CS is controlled by its amino groups, which, at low pH, are protonated and can thus interact with the negative change surface of mucin. Therefore, when these amino group are reduced as in the case of partially acetylated CS, its mucoadhesion is also drastically reduced [88]. In our case, during the synthesis of the derivatives, the radical polymerization starts from the amino groups and in both cases the number of amino groups is subsequently lowered. A reduction in the mucoadhesive strength should be expected accordingly. However, the existence of secondary amino groups as well as -SO_3_H groups in AAMPS monomer could lead to further increase of mucoadhesive strength. Likewise, in MEDPS monomer there are tertiary amino groups, which can explain the better mucoadhesive properties and also negative charge –SO_3_^−^, which could interact with the glycoproteins of mucus. Except for these interactions, it is well known that macromolecular interpenetration or diffusion into mucus are also responsible for enhanced mucoadhesion [78,89,90,91,92,93]. Amphiphilic monomers, such as 2-(methacryloyloxy)ethyl]-trimethylammonium chloride, have been used for copolymer synthesis with several methacrylates, and their mucoadhesion properties have been evaluated [94]. From this study, it was not clear how these groups behave, since hydrogen bonds or other electrostatic interactions were not formed between amphiphilic groups and mucin due to the absence of proton-donating and proton-accepting groups in their structures. However, it was found that as the content of hydrophobic groups and chain flexibility increases the mucoadhesion increases too, which proves the importance of hydrophobic effects in mucoadhesion. The chain flexibility was also reported to favour mucoadhesion in other studies [95,96]. So, it is possible that MEDPS, which is a larger molecular than AAMPS, has a higher interpenetration ability and physical entanglements with gel mucus molecules and thus higher mucoadhesive strength.

### 3.2. Characterization of Drug-Loaded Nanoparticles

**Figure 5:**
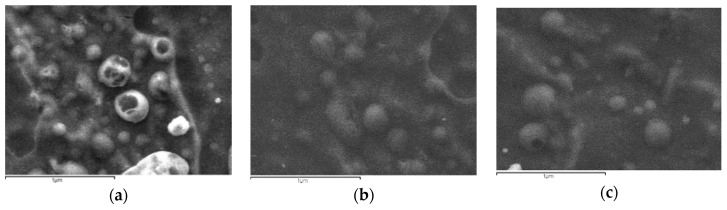
SEM micrographs of encapsulated drugs in (**a**) CS, (**b**) CS-AAMPS and (**c**) CS-MEDPS derivatives (scale bars are 1 μm).

The aim of this study was to prepare appropriate CS derivatives with mucoadhesive properties for the nanoencapsulation of CHL and DexSP drugs for ocular release formulations. As was already mentioned above, mucoadhesion mainly depends on the ability of a polymer to contact with mucus surface via several interactions. It was reported that this property is different when polymer is in bulk or in nanoscale [97]. Due to the high specific area of nanoparticles, the available interface for hydrogen bonding, ionic bonding or hydrophobic interactions with mucus surface increases dramatically. Ionic gelation is the most used technique to prepare nanoparticles of CS and its derivatives [55,98] and it was chosen in the present study as well. CS/TPP ration 4/1 was used since it gives nanoparticles with a low size diameter [99,100]. As can be seen in Figure 5, nanoparticles with a spherical shape have been prepared after the encapsulation of both drugs in CS and its derivatives. Comparing the nanoparticles formed by the different polymers, no clear difference can be observed in these SEM micrographs. However, a large size distribution is evident, since large particles with a diameter of about 500–800 nm and also much smaller nanoparticles of 100–200 nm are captured.

**Figure 6:**
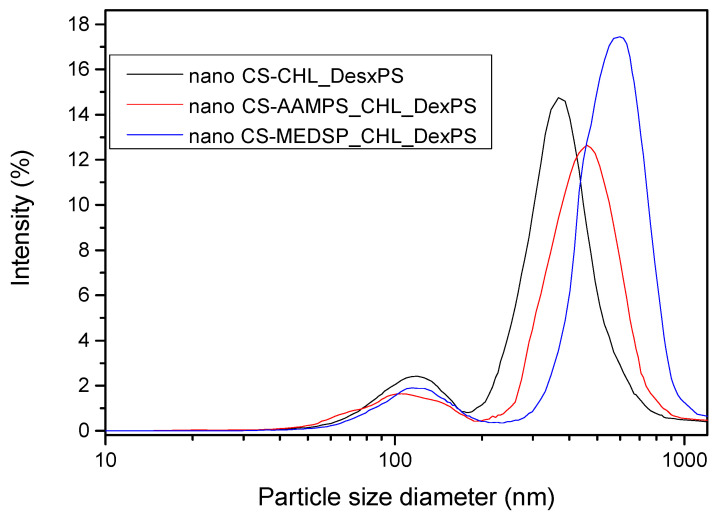
DLS curves of prepared nanoparticles with encapsulated CHL and DexPS drugs.

Drug-loaded nanoparticles with sizes ranging from 50 to 500 nm are considered ideal carriers for ocular drug delivery due to their ability to overcome ocular physiological barriers and diffuse rapidly to reach the epithelial lining [101]. Nanoparticles with size smaller than 500 nm have been reported to penetrate the mucin mesh [102,103] while those of a 1000-nm size are adhered to the mucus surface due to their inability to fit in these channels [104]. Larger particle sizes in the range of >1000 nm are being rapidly cleared from the mucus surface. SEM micrographs in combination with DLS data (Figure 6) allow us to presume that all prepared nanoparticles do not overcome the size of 1000 nm, and thus they could be appropriate for ocular drug delivery applications.

**Figure 7:**
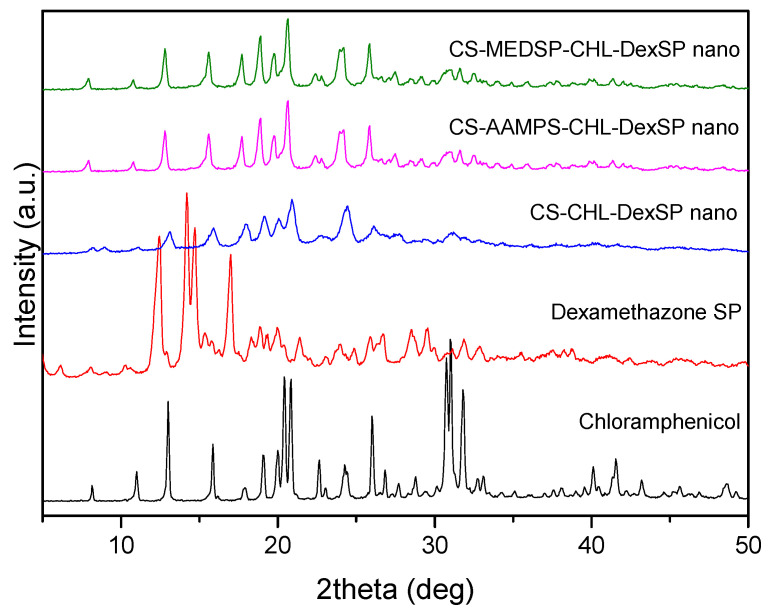
XRD patterns of neat drugs and encapsulated in CS, ACS-AAMPS and CS-MEDSP nanoparticles.

To verify the physical structure of encapsulated drugs in all used polymer matrices, XRD was used. As can be seen in the recorded patterns, both drugs are crystalline materials with several characteristic peaks (Figure 7). In the diffraction pattern of CHL, such peaks were recorded at 2θ: 8.18°, 10.98°, 12.96°, 15.86°, 17.81°, 19.05°, 20.01°, 20.41°, 20.84°, 22.64°, 24.26°, 26.01°, 26.84°, 27.69°, 28.80°, 30.75°, 31.02° and 31.79°, indicating that it is a high crystalline material. A similar picture was also observed in the DexSP diffraction pattern: four sharp peaks have been recorded at 2θ: 12.45°, 14.19°, 14.72°, and 17.02°, as well as some others of much lower intensity. In the diffraction patterns of nanoencapsulated drugs in CS and its derivatives, it is clear that only the CHL peaks have been recorded and these of DexSP are completely missing. This is an indication that DexSP was encapsulated in its amorphous form. Furthermore, comparing the peak position with these of the neat chloramphenicol drug, it can be seen that these peaks are broadening and also some new have been recorded. Taking into account that neat CS and its derivatives have no such peaks and only a very broad peak was recorded at 2θ about 20.3°, it is clear that these peaks are attributed to the chloramphenicol drug and are recorded almost at the same positions as in neat drug at 2θ: 7.94°, 10.79°, 12.80°, 15.61°, 17.69°, 18.91°, 19.77°, 20.62°, 22.37°, 24.14°, 25.84°, 26.64°, 27.49°, 28.52°, 28.94°, 30.94° and 31.61°. Some small differences, such as the upset of the peak at 20.84° and the recorded peaks after 27° of lower intensity than in the initial used drug, are evidence that the crystal structure has been slightly changed after nanoencapsulation. Moreover, the peak broadening indicates that some drug was encapsulated in the amorphous form inside the nanoparticles. However, since the characteristic diffraction peaks of DexSP were not recorded, the question was whether the drug was encapsulated in amorphous form or due to its high solubility it was unable to be encapsulated remained to be answered.

**Figure 8:**
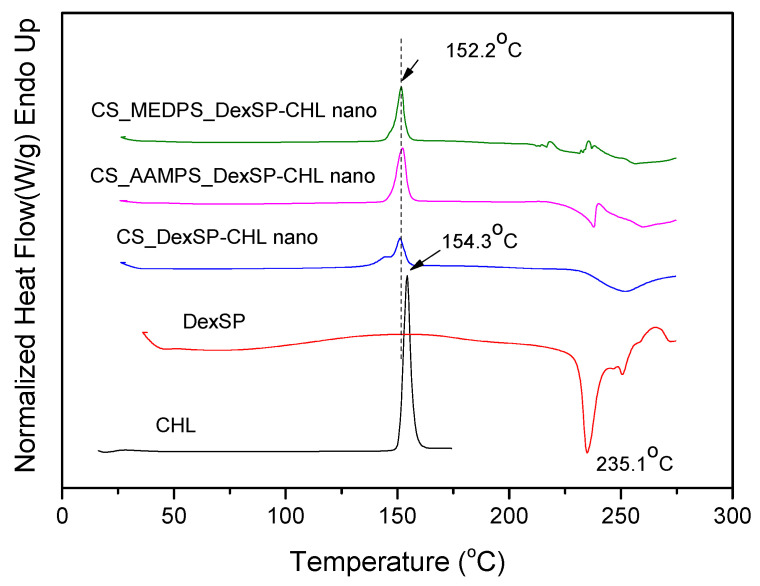
DSC thermograms of neat drugs and encapsulated in CS, ACS-AAMPS and CS-MEDSP nanoparticles.

**Figure 9:**
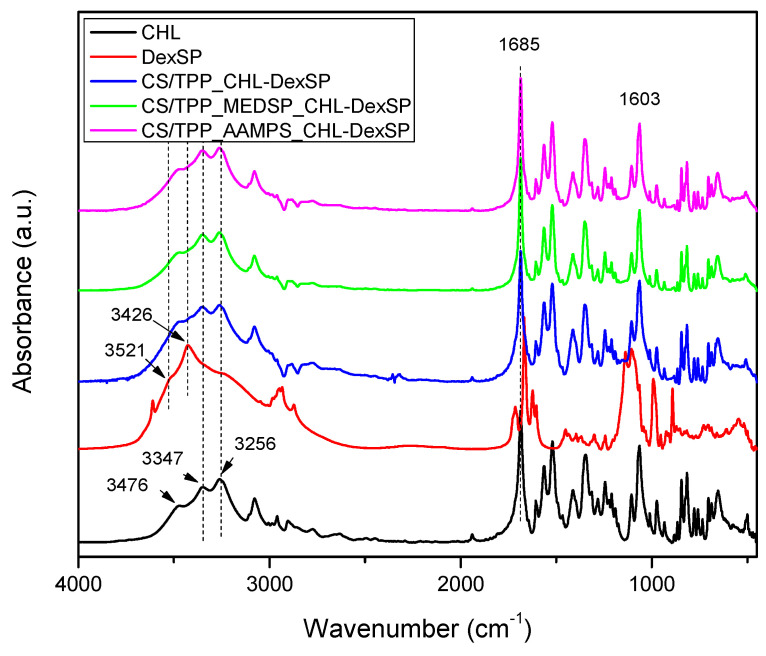
FT-IR spectra of neat drugs and encapsulated in CS, CS-AAMPS and CS-MEDSP nanoparticles.

In order to confirm the crystalline or amorphous nature of encapsulated drugs in nanoparticles, DSC thermograms have been also recorded. As can be seen in Figure 8, CHL has a melting point at 154.3 °C, which is very close to that reported from Khan et al. (around 150.48 °C) [105]. However, in DexSP thermograms, there is no melting point recorded, but only an exothermic peak with a maximum at 235.1 °C. This is because DexSP decomposes during melting and such decomposition was also mentioned in literature [106,107]. In drug nanoencapsulated materials, there is a clear melting point at about 152.2 °C, and an exothermic peak started from 220 up to 250 °C, which are attributed to encapsulated CHL and DexSP drugs, respectively. From these thermograms, it was confirmed that both drugs have indeed been encapsulated in nanoparticles, which, in the case of CHL, was also proved from XRD. The exothermic peak in all encapsulated nanoparticles is clear proof that DexSP was encapsulated in nanoparticles and probably in its amorphous form, since on its XRD patterns its crystalline peaks have not been recorded. The differences in recorded positions such as in lower T_m_ temperature for CHL and in higher decomposition temperatures for DexSP, compared with neat drugs, is an indication that maybe some interactions took place between the polymer matrices and drugs. However, this is not clear, and maybe these differences arise from the fine dispersion of both drugs in polymeric nanoparticles. To evaluate the existence of such interactions, the FT-IR spectra of both neat drugs and their nanoparticles (Figure 9) were obtained by FT-IR spectrophotometer using potassium bromide (KBr) pellets in the area of 4000 to 400 cm^−1^.

The CHL drug showed the main characteristic peaks attributed to the functional groups on its molecule, such as the –OH stretching vibration at 3476, 3347 and cm^−1^, the –N–H stretching at 3259 cm^−^^1^, and the peak at 3081–2927 cm^−1^ (maximum at 3076 cm^−^^1^), which was assigned to the aromatic C–H stretching. The stretching vibrations at 1685 and 1606 cm^−1^ is due to CO–NH amide stretching and vinyl groups, respectively. The peak at 1563 cm^−1^ is owing to –NO_2_ asymmetric stretching (ArNO_2_) and at 1520 cm^−1^ the N–H bending was recorded. The peak at 1346 cm^−1^ is due to C=O stretching mode, the band at 1260 cm^−^^1^ is due to C–O stretching vibration and the band region at 1242 cm^−^^1^ has been assigned to C–N symmetry stretching. At regions 1063 cm^−1^ and 975 cm^−^^1^ the C–O stretching absorption of primary alcohol and secondary alcohol, respectively, are recorded while the peak at 847 cm^−1^ is due to C–N stretching (out of plane NH-bending). Finally, the C–Cl stretching was recorded at 653 cm^−1^.

The FT-IR spectrum of DexSP showed a small intensity and narrow peak at 3613 cm^−^^1^ (due to free –OH groups), a broad peak with two maxima at 3521 and 3426 cm^−^^1^ due to O–H stretching vibration, the peaks at 1715 and 1668 cm^−^^1^ are assigned to the vibration of the conjugated (a) and unconjugated (b) carbonyl groups, respectively, the peaks at 1624 and 1604 cm^−^^1^ refer to vinyl groups’ C=C stretching vibrations, and the peaks at 1135–1104 cm^−^^1^ are due to CO stretching. The stretching frequency bands of phosphate anion (P–O) are recorded at 1062 and 1012 cm^−^^1^, while the band at 992 cm^−^^1^ is corresponded to P–O–C group stretching and deformation vibrations.

Comparing the spectra of neat drugs and the encapsulated in CS and its derivatives, it can be seen that almost all the characteristic peaks of the drugs remain in the same positions without any shift. Even though both drugs have –OH groups and carbonyl groups and some interactions with the amino and sulfonic groups of CS and their derivatives would be expected, it seems that such interactions are not taking place. This may be because some intermacromolecular interactions between the reactive groups in neat CS and their derivatives are already present. This would be expected mainly in the case of CHL since, from XRD patterns and DSC thermograms, it becomes apparent that the drug was encapsulated in crystalline form. Usually, when interactions are taking place between a polymer and a drug, as in the case of hydrogen bonding between chloramphenicol and poly(vinyl pyrrolidone) (PVP), poly(vinyl alcohol) and Eudragit^®^ [108], amorphous dispersions are produced. In the case of dexamethasone, some interactions with polymers through hydrogen bonding or ionic bonds between PO_4_^3^^−^ and –N(R)_2_H^+^ have been reported [109], as well as through intercalation into layered double hydroxides (LDHs) in which strong electrostatic interactions have been detected [110]. However, such interactions have not been detected in our nanoparticles and the drug amorphization should be due to the high DexSP solubility and probably due to its fine dispersion into the nanoparticles.

### 3.3. Drug Release

The aim of the present work was to enhance the solubility of CHL and also to prepare mucoadhesive nanoparticles for extended ocular release of both CHL and DexSP drugs. This is necessary in order to enhance the bioavailability of the poorly water soluble CHL and to improve the effectiveness of both drugs by increasing their residence time in eye surface. In the literature, there are limited works for simultaneous administration of both CHL and DexSP drugs. In such an attempt, gellan gum, which is an ion-sensitive polymer, was used with poly(acrylic acid) (carbopol 940) to prepare an in situ ophthalmic gel for simultaneous sustained release of both drugs [111]. These matrices have also high mucoadhesive strength and it was found that by increasing the polymer concentration in solutions, the drug release was decreased due to the higher entangled ability of the polymeric network. The solution concentrations consisted of 0.1% DexSP and 0.5% (*w*/*v*) CHL and it was reported that the CHL drug was completed released within 12 h, while the release of DexSP was retained up to 10–12 h. However, this release time is too short and should be further extended in order to reduce the required daily repeated administration. Such extended release times seem to be achieved in the present study.

The yield, as well as the drug loading, of all prepared nanoparticles were calculated, and the results are presented in Table 3. It can be viewed that the yield of nanoparticles is ranging between 40 up to 55%. CS scores the highest yield, whereas its derivatives give lower values. A nice explanation for that would come from the fact that the CS derivatives contain a much lower number of free amino groups to react with TPP in order to form the nanoparticles. Moreover, it is possible that the grafting of AAMPS and MEDPS groups increases the distance between nearby macromolecules (due to the size of the grafted monomers), and therefore the ability of their amino groups to interact with TPP ionic groups is inhibited. Concerning the drug loading, the differences between all nanoparticles are insignificant in order to export any clear trend or conclusion. However, a higher loading content of CHL is achieved in all cases, compared to DexSP. This should be attributed to the higher solubility of DexSP. The entrapment efficiency or percentage of the content of each drug was estimated, showing that about 25 to 30% of initial weighted CHL was successfully incorporated in the nanoparticles, while the corresponding value for DexSP was varied between 22 and 27%, without following any specific trend.

**Figure 10:**
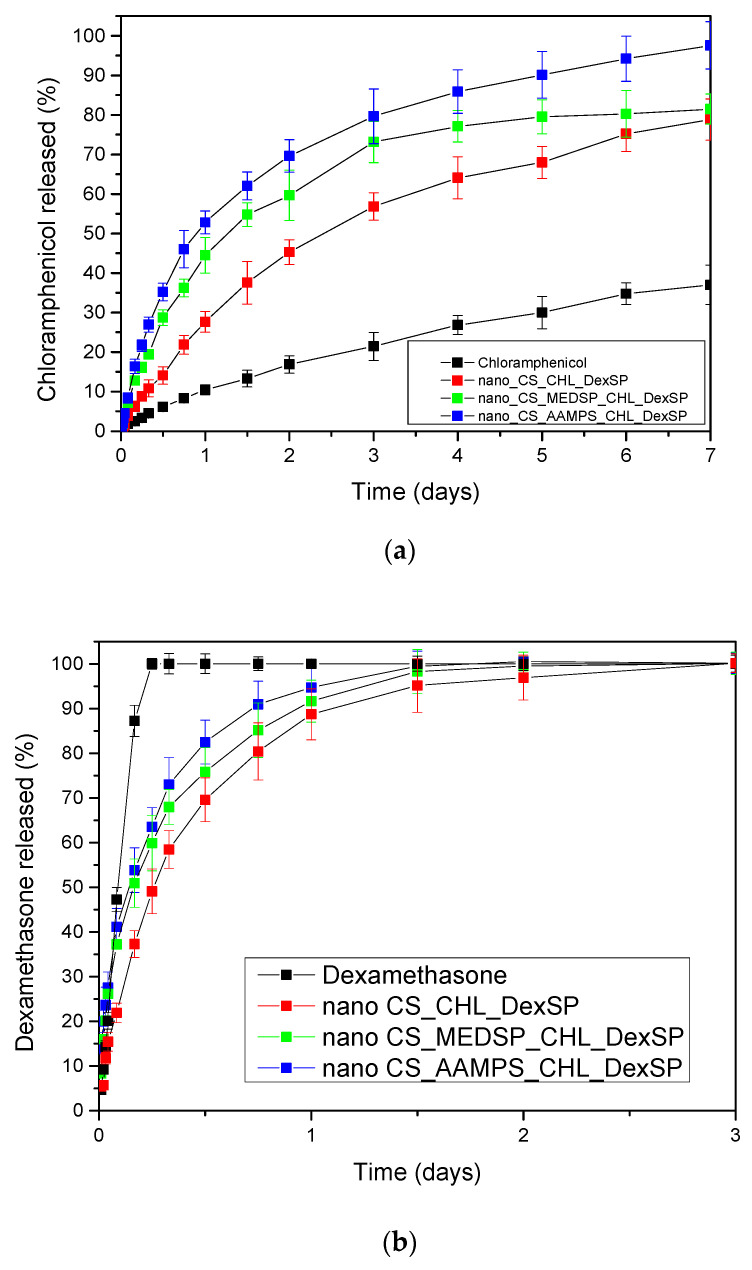
In vitro release of (**a**) CHL and (**b**) DexSP from prepared nanoparticles at pH = 7.4.

In Figure 10, the in vitro release profiles of both CHL and DexSP drugs from all prepared nanoparticles are presented. As can be seen, the drugs, due to their different solubilities, have different release profiles. CHL is a poorly soluble drug and has a much lower and extended release, while DesSP is a freely water-soluble drug and, for this reason, has an almost immediately release profile. As can be seen in Figure 10a, neat CHL has a low solubility, even after 7 days, which reaches about 37%. In all prepared nanoparticles, this solubility was enhanced and was close to 80% for CS and CS-MEDSP nanoparticles and about 98% from CS-AAMPS nanoparticles. In all cases, there is a biphasic drug release since they have an initial burst release followed by a sustained release, which is very common in nanoparticles. The rapid release (within 1.5 h) is thought to be associated with the drug that is absorbed or weakly linked to the surface area of the nanoparticles, while the extended release is associated with the release of the residual quantity of the drug that is mostly entrapped in the nanoparticles. However, these release profiles clearly suggest that CHL-loaded nanoparticles can enhance the drug solubility, compared to neat CHL, which was one of the aims of this study. Suchlike enhancements have been reported in literature using sulfobutylether-β-cyclodextrin/chitosan nanoparticles for ocular drug delivery [112], CDs’ inclusions [31] and their derivatives with sulfonic groups, such as the sulfobutyl ether-β-cyclodextrin (SBE-β-CD) [113]. It was found that SBE-β-CD has a higher complexation efficiency than neat CD, and a 1:1 inclusion complex was formed, in which chloramphenicol was entrapped in amorphous form. Furthermore, it was found that this derivative is more appropriate than neat CD, since CHL release could be extended from the SBE-β-CD inclusion complex over a period of 5 h. This was also proved by in vivo studies in rabbits, where CHL inclusion in SBE-β-CD showed much longer residence time in conjunctival sac and thus higher ocular bioavailability. Comparing these release profiles with our study, it is pronounced that nanoparticles that can further extend the release time up to 7 days were successfully produced. Moreover, there is an apparent connection between the release profile and the polymer used during nanoparticles preparation: CS nanoparticles are characterized by much slower release rates, whereas CHL exits the CS-AAMPS nanoparticles in a much faster way. This could be attributed to the high swelling properties of CS-AAMPS (Figure 4) allowing the drug to be easily released, while the lowest swelling properties of CS hinder the release. These prolonged release findings are clearly in agreement with the reported literature using several nanoparticles [112] as well as chitosan-gelatin hydrogels, which showed promising mucoadhesive and in vitro release rates for a longer period of time for the timolol drug, compared to the conventional eye drops [114]. It seems that hydrogels may be ideal systems for extended and controlled release applications for CHL drug [115], and our prepared derivatives behave much alike. Furthermore, the dissolution profiles of the current study marked even higher rates than solid dispersions of chloramphenicol in PEG8000, which have been prepared to enhance the dissolution rate of CHL but still only a maximum of 60% of drug release was achieved [105]. 

The release rates of DexSP is completely different for all nanoparticles compared to that of CHL. There are three distinguished release stages. In the first stage, an accelerated and almost immediate release can be seen, known as the “burst effect”, which is followed by a slower and more controlled release stage, and after that a very slow release reaching almost a plateau is recorded. However, in all formulations, complete drug administration is achieved lasting up to 3 days. The initial burst release is much greater here than that in the case of CHL, and almost the 35–40% of the drug amount was released in the first 2 h. This is due to the high solubility of DexSP (almost 90% of the neat drug is now soluble) and, for this reason, the drug amount that was partially adsorbed onto the nanoparticles’ surface is immediately released. After this stage, the DexSP trapped inside the nanoparticles was relatively slowly released, but in higher rates compared to CHL. After 24–36 h, the slowest release and a resulting plateau are recorded. Comparing the different polymers, it may be observed that CS nanoparticles again gave the lowest release rate, as in the case of CHL. This could be probably attributed to its lowest degree of swelling or due to the strongest interactions of CS with both drugs. Concerning the two CS derivatives, even though the differences in their release rates are not remarkable enough, it seems that the release is again faster in the case of CS-AAMPS derivative as in the corresponding CHL-loaded nanoparticles. Yet, the exact opposite was expected here.

MEDSP contains tertiary amino groups that could ionically interact with the phosphate groups of DexSP, leading to a delayed drug release. Such slower release was reported in the quaternary ammonium chitosan derivative that was prepared with hydroxypropyltrimethyl ammonium chloride (HACC) and nanostructured lipid carriers (NLC) [116]. It was found that the release rate of Dex from NLC-HACC hydrogels was slower than that from NLC. However, in our case, it seems that the swelling ratio of the prepared derivatives, and not the charge of the used carriers, plays the most important role. This was also studied in a recent work in which lipid nanoparticles (L/NPs) with negative (−) and positive (+) surface charges have been tested to evaluate if the different surface nature affects dexamethasone release in vivo [117]. Even though the CS-modified L/NPs yielded a more sustained and controlled release of Dex, compared to the unmodified L/NPs, both (−) or (+) L/NPs exhibited higher dexametha-sone bioavailability (*P* < 0.05) to rabbits’ eyes, as compared to the aqueous solution (2.12- and 4.69-fold higher, respectively), while the difference between them was negligible. The respective *T*_max_ values of DEX from (+) and (−) L/NPs were 12- and 10-fold higher, whereas the respective *C*_max_ values were 2.37- and 1.17-fold higher, respectively, than the values for the aqueous solution.

Due to the high DexSP solubility, it was not possible to achieve a sustained release profile as in the case of CHL. Such long-acting formulations for DexSP administration can be prepared only when synthetic, non-swellable polymers are used. Such examples have been reported in literature using poly(ethylene glycol) (PEG) with zinc ion bridging [118] for DexSP nanoencapsulation and PLGA microspheres [119,120] where DexSP was released up to 25 and 30 days, respectively. Similar extended release profiles of Dex have been reported for intracanalicular applications (up to 30 days) [121] and up to 12 days from poly(ɛ-caprolactone) nanofibers [122]. However, these polymers have low mucoadhesion and thus cannot be used in drop eyes solutions. 

### 3.4. Modeling of Drug Release Data

In this section, the drug release behaviour that has been qualitatively discussed before will be analysed through quantitative means. The release mechanism is quite different in the case of pure drugs than in the case of drug-loaded nanoparticles. The mechanism for pure drug is dissolution (i.e., transition of drug from solid to liquid phase), whereas for nanoparticles is diffusion in the polymer matrix. 

**Figure 11:**
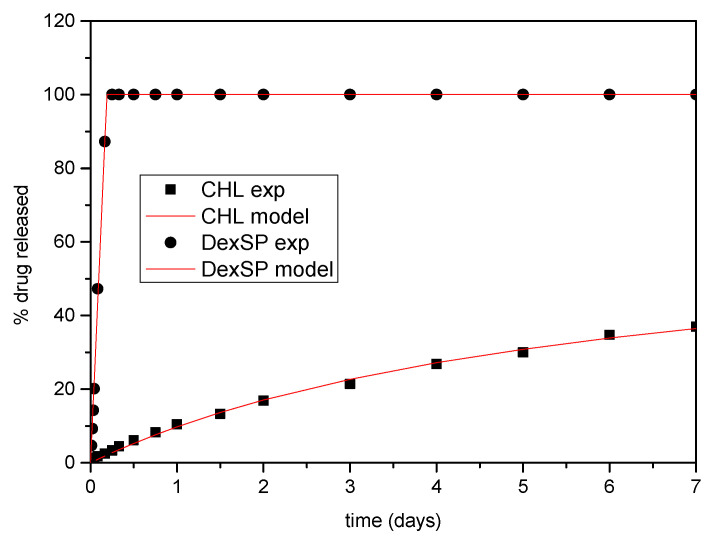
Comparison between model (solid lines) and experimental (symbols) release curves for pure drugs.

At first, the pure drug release kinetics will be examined. It is noted that primitive models, such as the Noyes–Whitney equation [123] cannot describe the present data, and, consequently, more sophisticated models are needed. The release kinetics consist of two steps: phase change and mass transfer the boundary layer close to the solid and the bulk liquid. It is assumed here considering the characteristic release time (more than 4 h) and the agitation of the system that the mass transfer process does not set kinetic limitations. So, the release of the pure drugs is dominated from the phase change, which can be considered as a surface reaction of order n. The reaction rate is k(c_eq_-c)^n^ (units of mass per second), where c_eq_ is the equilibrium concentration of the drug in the liquid (i.e., solubility) and c is its instantaneous concentration. The idea is that the phase change is as faster as larger is the difference between the actual and the equilibrium concentration. Performing a drug mass balance in the liquid leads to $V\frac{dc}{dt}=k{\left(c_{eq}-c\right)}^{n}$, where V is the liquid volume. The release R% can be computed from c as R% = 100 Vc/m, where m is the initial mass of the drug used in the experiment. The above mathematical problem can be integrated and finally leads to:

(6) $$R\%=R_{\max}[1-{\left(1-(1-n)At\right)}^{1/(1-n)}]$$

where R_max_ is the maximum release and $A=\frac{kc_{eq}^{n-1}}{V}$. Obviously, both R_max_ and R% cannot overcome the value of 100. The above equation is fitted to the experimental release data. In case of DexSP, the R_max_ is clearly 100, so the fit was made with respect to n and A. In the case of CHL, the fit was made with respect to R_max_, n, A. It came up that a phase change reaction of zero order holds for DexSP and of second order holds for CHL. The values n = 0 and n = 2 found for the two drugs is a confirmation that the mass transfer does not contribute to the release kinetics since its corresponding value is n = 1. In addition, it is clear why the Noyes–Whitney equation (which also corresponds to n = 1) cannot represent the data for the particular drugs examined. It is noticed that the fit to the experimental data is extremely successful, having a root mean square value of the difference between experimental and model data less than 1% of release. This deviation can be attributed to the scatter of experimental data so it can be considered that the zeroth and first order rates fully describes the release of DexSP and CHL respectively. The comparison between experimental data and model curves is shown in Figure 11. The other parameter values are A = 5.26 1/day for DexSP and A = 0.17 1/day, R_max_ = 67 for CHL.

The modeling of release from nanoparticles is the subject of what follows. The swelling kinetics are too fast (characteristic time at about 1 h) to have any effect on the much slower release process. There are several empirical expressions for diffusion-dominated release, but, typically, they have a power law form and they are restricted to limited release (far from completion) [124]. In case of the full extent of the process and Fickian diffusion, the transient diffusion equation has a series solution in terms of its eigenvalues and eigenfunctions. An alternative to this solution is an approximate formula based on assumed parabolic profile of drug loading in the particle during the whole process. This formula is called linear driving force approximation [125] and finally leads to a single exponential form of the release curve:

(7) $$R\%=R_{\max}(1-\exp(-Kt))$$

**Figure 12:**
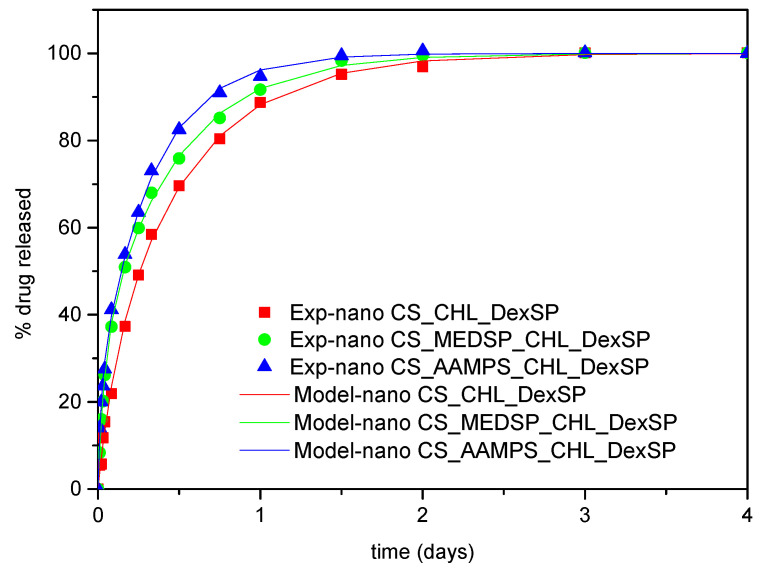
Comparison between model (solid lines) and experimental (symbols) release curves for DexSP in three types of nanoparticles.

**Figure 13:**
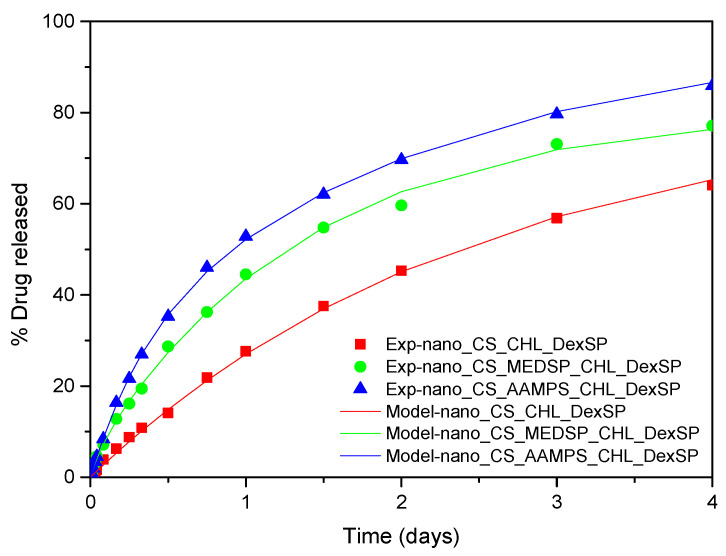
Comparison between model (solid lines) and experimental (symbols) release curves for CHL in three types of nanoparticles.

The limiting value R_max_ in this case is not related to the drug solubility in the liquid as in case of pure drugs but to the mobility of the drug in the nanoparticles. A value of R_max_ smaller than 100 implies a portion of drug immobilized in the nanoparticle (i.e., by exhibiting a crystallinity degree). The parameter K is related to physical parameters as K = 15D/r^2^, where D is the diffusivity of drug in nanoparticle and r is the nanoparticle radius. The attempt to fit the data using the above equation was unsuccessful. The fitting could be perfect at a small time region or at a large time region but no at the whole time domain. The type of the deviation led to adding another exponential term in Equation (6). The proposed equation has the form:

(8) $$R\%=R_{\max}[1-\varphi \exp(-K_{1}t)-(1-\varphi )\exp(-K_{2}t)]$$

where φ is the fraction of the mobile drug that is released with a kinetic constant K_1_ and 1-φ is the fraction released with kinetic constant K_2_. The fitting of the data was very successful using Equation (8). The root mean square of the difference between data and model values is at about 1% of release. The comparison between experimental data and model curves for all the type of nanoparticles and both drugs employed appear in Figure 12 and Figure 13. The fitting values for the parameters φ, K_1_, K_2_ appear in Table 4.

A question arises on what the physical origin of the above model is. There are several possible answers. The first is that the exact solution of the diffusion equation can be written as an infinite sum of exponentials so this is a possible explanation for Equation (8). However, in this case, the ratio K_2_/K_1_ should always be equal to 5 (ratio between the first two eigenvalues of the diffusion equation [126]). Obviously, this is not what happens here, so this possibility is rejected. Another possible source is the bimodality of the particle size distribution. This can also support the double exponential release curve. However, performing a volumetric analysis appears that the smaller mode contains only about one percent of the total drug so it is not compatible to the values of φ appearing in the Table 4. A third possibility, which is the most probable one, is a non-uniform distribution of the drug in the nanoparticle. The portion φ is close to nanoparticle surface and has direct access to the liquid, leading to high release kinetics, whereas the portion 1-φ diffuses slowly from the interior of the nanoparticle to its surface. Employing K_2_ values and volumetric average particle radii the diffusion coefficient of the drug in the nanoparticle can be estimated. The values of D for DexSP were found to be in the range 4.5 × 10^−20^–2.5 × 10^−19^ m^2^/s and for CHL to be in the range 1 × 10^−20^–3 × 10^−20^ m^2^/s. These values are typical for the diffusion of drug molecules from polymeric matrices. It is clear that drug diffusibility from all polymer matrices is larger for DexSP than for CHL. Finally, for both drugs, the matrix composition affects the diffusivity, which increases in the order CS_AAMPS > CS_MEDSP > CS_CHL.

## 4. Conclusions

CS derivatives with 2-acrylamido-2-methyl-1-propanesulfonic acid and 2-(methacryloyloxy)ethyl]dimethyl-(3-sulfopropyl)ammonium hydroxide have been prepared and studied for the first time for ocular release formulations. The advantage of these derivatives is that, due to their functional groups, they have enhanced antimicrobial and mainly mucoadhesive properties, which is very important to prolong the residence time of solution drops in the ocular surface. Furthermore, these derivatives have a high degree of swelling, an essential property that allows them to strongly adhere to the mucus surface and controls the drug release rate. CHL- and DexSP-loaded nanoparticles in CS and its synthesised derivatives, have been successfully prepared via ionic gelation with TPP. They had spherical shapes and particles sizes of less than 1000 nm, which is a significant factor, since such low sizes can penetrate the mucus surface, and consequently increase the drug effectiveness. The bioavailability of the poorly water drug CHL was substantially enhanced in all studied nanoparticles. Drug release was apparently dependent upon the used polymer and was higher in the case of CS-AAMPS nanoparticles, owing to the greater degree of swelling of the derivative, while CS had the lowest rate, due to its low degree of swelling. The release rate of DexSP, which is a freely water-soluble drug, was much extended for all prepared nanoparticles up to 3 days. From drug release modelling, it was found that diffusion is the main release mechanism for both drugs. The diffusion rate is slightly higher in DexSP than CHL, while diffusivity increases in the order CS_AAMPS > CS_MEDSP > CS_CHL. Based on drug release profiles, it is clear that all nanoparticles are appropriate for extended and simultaneous ocular release formulations of DexSP and CHL drugs, ranging from at least 3 to up to 7 days.

## Figures and Tables

**Table 1:** Inhibition growth zones of CS and its derivatives against the studied strains, compared with the used monomers and neomycin.

Polymer	Area of Inhibition (Diameter mm)	
	*E. coli*	*S. aureus*
CS	6 ± 1	7 ± 1
CS-AAMPS	7 ± 1	12 ± 2
AAMPS monomer	8 ± 1	13 ± 2
CS-MEDSP	9 ± 2	14 ± 3
MEDSP monomer	8 ± 1	13 ± 2
Neomycin	12 ± 2	10 ± 1

**Table 2:** Mucoadhesive strength of CS and its derivatives.

Polymer	Mucoadhesive Strength (N/cm^2^)
CS	0.27 ± 0.02
CS-AAMPS	0.46 ± 0.05
CS-MEDPS	0.72 ± 0.07

**Table 3:** %yield of nanoparticlesand %drug loading.

Sample	Yield (%)	Drug Loading (%)		Entrapment Efficiency (%)	
		CHL	DexSP	CHL	DexSP
CS/TPP	55.1 ± 5.4	23.7 ± 2.1	10.1 ± 0.6	29.28 ± 2.3	24.96 ± 2.7
CCS/TPP-AAMPS	46.8 ± 3.7	25.2 ± 1.9	9.7 ± 0.6	28.78 ± 1.5	22.15 ± 1.4
CS/TPP-MEDSP	43.5 ± 3.8	22.6 ± 1.5	11.8 ± 0.8	25.98 ± 1.9	27.13 ± 1.5

**Table 4:** Parameter values for fitting the experimental release data using Equation (4).

Drug	Matrix	φ	Κ_1_(1/day)	Κ_2_(1/day)	R_max_
DexSP	CS	0.21	10.28	1.9	100
DexSP	CS-MEDSP	0.31	23.18	2.15	100
DexSP	CS-AAMPS	0.23	49.5	3	100
CHL	CS	0	-	0.14	81.7
CHL	CS-MEDSP	0.05	13.34	0.72	80.5
CHL	CS-AAMPS	0.28	2.9	0.46	97.5
